# Prevention of Adolescent Problem Behavior: Longitudinal Impact of the Project P.A.T.H.S. in Hong Kong

**DOI:** 10.1100/tsw.2011.33

**Published:** 2011-03-07

**Authors:** Daniel T. L. Shek, Lu Yu

**Affiliations:** ^1^ Department of Applied Social Sciences, The Hong Kong Polytechnic University, Hong Kong; ^2^ Public Policy Research Institute, The Hong Kong Polytechnic University, Hong Kong; ^3^ Department of Social Work, East China Normal University, Shanghai, China; ^4^ Kiang Wu Nursing College of Macau, Macau, China; ^5^ Department of Pediatrics, University of Kentucky College of Medicine, Lexington, KY, USA

**Keywords:** adolescent problem behavior, longitudinal study, positive youth development, prevention, Project P.A.T.H.S., randomized group trial

## Abstract

The present study attempts to examine the longitudinal impact of a curriculum-based positive youth development program, entitled the Project P.A.T.H.S. (Positive Adolescent Training through Holistic Social Programmes), on adolescent problem behavior in Hong Kong. Using a longitudinal randomized group design, six waves of data were collected from 19 experimental schools (n = 3,797 at Wave 1) in which students participated in the Project P.A.T.H.S. and 24 control schools (n = 4,049 at Wave 1). At each wave, students responded to questions asking about their current problem behaviors, including delinquency and use of different types of drugs, and their intentions of engaging in such behaviors in the future. Results based on individual growth curve modeling generally showed that the participants displayed lower levels of substance abuse and delinquent behavior than did the control students. Participants who regarded the program to be helpful also showed lower levels of problem behavior than did the control students. The present findings suggest that the Project P.A.T.H.S. is effective in preventing adolescent problem behavior in the junior secondary school years.

## INTRODUCTION

Adolescent problem behaviors, such as alcohol use, delinquency, teenage pregnancy, violence, and different types of substance abuse, are always of concern to health professionals and researchers. In the past 2 decades, the strategy to prevent adolescent problem behaviors has evolved from problem monitoring to the use of strength-based approaches that focus on the promotion of positive youth development[1]. Researchers have pointed out that problem-oriented programs did not engage the motivations and capacities of young people, failed to address complicated personal and social antecedents of problem behaviors, and did not consider youth development as a gradual and cumulative process in which most youth would become productive and caring adults despite experiencing risks and challenges[2,3,4]. Based on these arguments, the notion of positive youth development has been proposed, which emphasizes adolescents' resilience, strengths, and potential contributions to others as well as to society, and advocates that youth problematic behaviors could be more effectively prevented by promoting positive youth development, such as prosocial behaviors, trusting relationships, positive self-identity, a sense of hope, social competence, academic performance, and resilience[2].

Based on this strength-based view, a number of positive youth development programs have been designed and implemented[5,6]. Evaluative studies of the programs in the field have provided support for the effectiveness of fostering youth developmental assets[7] in reducing problem behaviors[2,8,9]. For example, it was reported that after attending a school development program focused on enhancing youths' attachment to school, participants' risk behaviors (e.g., academic failure, alienation, and association with drug-using peers) diminished, while their health-promoting behaviors increased[10]. In the Communities That Care (CTC) program, researchers examined the long-term effects of an intervention that incorporates parental education, teacher training, and social competence training on adolescent health risk behaviors for elementary school students living in high-crime communities[11]. By age 18, students who received the full CTC intervention reported fewer numbers of problem behaviors, including violence, sexual intercourse, multiple sex partners, delinquent behaviors, heavy drinking, and pregnancy, and they had greater commitment and attachment to schools and better academic achievement than did students in the control groups. In a systematic review, Catalano and colleagues identified 25 rigorously evaluated positive youth development programs that demonstrated significant positive program effects in decreasing adolescent risk behaviors either immediately or over time[2].

Nonetheless, most of the positive youth development programs are developed and carried out in the West, particularly the U.S. In the context of Asia, a survey of the literature shows that there are very few programs that address adolescent problem behaviors using the positive youth development approach despite the proven effectiveness of such programs in Western studies[12]. Among the limited number of youth programs, rigorously evaluated programs are even more lacking. With specific reference to Hong Kong, which is a more Westernized and developed society, although researchers have warned that adolescent problem behavior, such as smoking, drinking, substance abuse, Internet addiction, and pathological gambling, is rising[13], well-designed positive youth development programs with a systematic evaluation component are almost nonexistent.

Against this background, Shek and researchers from five Universities in Hong Kong designed and implemented a large-scale youth enhancement program entitled the Project P.A.T.H.S. (**P**ositive **A**dolescent **T**raining through **H**olistic **S**ocial Programmes), which aims to promote positive development among Hong Kong adolescents and reduce their risk/problem behaviors[14,15]. There are two tiers of programs in the Project P.A.T.H.S. Both tiers are developed with reference to 15 positive youth development constructs, including bonding, resilience, social competence, recognition of positive behavior, emotional competence, cognitive competence, behavioral competence, moral competence, self-determination, self-efficacy, clear and positive identity, beliefs in the future, prosocial involvement, prosocial norms, and thriving. The project has been implemented in more than 250 schools in Hong Kong[2].

The Tier 1 Program is a universal positive youth development program in which students in Secondary 1 to 3 take part. There are 20 h of training in both core and elective programs each school year for each grade. Because research findings suggest that roughly one-fifth of adolescents would need more help, the Tier 2 Program is provided for those students who display greater psychosocial needs at each grade (at least one-fifth of the students). The programs in both tiers can also be used in the counselling and guidance contexts of schools.

The Tier 1 Program has several characteristics. First, the number of hours for each grade of the junior secondary school is 20. There are core units of 10 h and elective units of another 10 h. For schools with special or extra needs, they may choose the core units only (i.e., 10 h for the Tier 1 Program). In any case, the minimum number of hours for the Tier 1 Program in a school should be 10 h. Second, as there are 40 units per grade (each lasts for 30 min), students will have completed a total of 120 units by the time they finish the three junior secondary school years. The units in the program are constructed with reference to the positive development constructs described earlier and identified in the successful programs. Third, the program was developed by the research team by integrating existing research findings, programs, local adolescent needs, cultural characteristics, and experiences gained from the Experimental Implementation Phase of the Project. Trial teaching for all units was carried out. Fourth, relevant adolescent developmental concerns (e.g., drug issues, sexuality, financial management, sense of responsibility, life meaning) and adolescent developmental strengths (e.g., high level of concern for society and high proficiency in information technology) were incorporated into the program. To cater to the needs of different schools, different activities were designed.

The fifth characteristic of the program is that its effectiveness is maximized to the students. To achieve this, the Tier 1 Program is preferably run by both a teacher and a social worker. If this ideal cannot be attained, a teacher or a social worker can run the program on his/her own, but preferably with the assistance of a helper. To facilitate implementation, the program was designed in such a way that one teacher or social worker can implement it without much difficulty under normal circumstances. In terms of qualifications, the social worker mainly responsible for implementing the Tier 1 Program should be registered, preferably with a university degree and at least 2 years of social work experience. Finally, systematic and adequate training is another emphasis of the program. For each of the Secondary 1 to 3 programs, both teachers and social workers involved receive 20 h of training before the implementation.

An important feature of the Project P.A.T.H.S. is its systematic evaluation approaches (e.g., interim evaluation, focus-group interview, survey on subjective and objective outcomes, program implementers' evaluation, student weekly diary, etc.), which enable researchers to examine the effectiveness of the program thoroughly[16,17]. To delineate the process of how the Project P.A.T.H.S. may have causal effect on the developmental trajectory of adolescent positive and negative outcomes, Shek and his research team have designed and implemented a longitudinal randomized group trial, including 24 experimental schools and 24 control schools recruited at the initial period of the project. The first two waves of data collected in the longitudinal study showed that participants in the experimental group exhibited greater improvements in different positive youth development constructs at post-test than did the control group students[17]. Based on the first four waves of data in the trial, it was found that students who participated in the program had significantly better positive outcomes in terms of psychosocial competence, academic and school behavior, and global positive youth development, while they exhibited lower levels of delinquent behaviors as compared to students in the control group[18].

It is noteworthy that generalized linear models are commonly used to examine effectiveness of adolescent prevention and positive youth development programs over time. However, there are views arguing that generalized linear models, such as analyses of covariance, only estimate and compare the group means and are not informative about individual growth[19]. It has been advocated that more advanced statistical methods, such as Hierarchical Linear Modeling (HLM) or Latent Growth Curve Modeling (LGCM), should be used to investigate program impacts on participants over time. In such models, repeated measurements on the same subjects are viewed as a separate level nested within an individual, and structured covariance matrices and the parameters in a regression model are allowed to vary across individuals. In addition, time-varying or other individual factors could be added to the model as covariates, which would allow researchers to estimate individual growth curves precisely. Besides, unlike the traditional analyses of variance, HLM and LGCM do not require every participant to have the same number of observations and thus are capable of handling longitudinal data with unbalanced structure.

Using linear mixed models via SPSS, Shek and Ma[19] investigated the effects of the Project P.A.T.H.S. on participants' positive development based on six waves of data collected in the trial and found that participants in the experimental schools displayed better positive youth development in terms of positive self-identity, prosocial behavior, and general positive youth development attributes. While the findings reported by Shek and Ma[19] are pioneering, there is a need to examine the impact of the project on the problem behaviors of the participants over time. Therefore, the purpose of the present study was to examine the longitudinal effects of the Project P.A.T.H.S. on the developmental trajectory of youth problem behaviors. Based on previous studies, it was hypothesized that participants in the experimental group would display less negative developmental outcomes than the control participants in terms of different problem behaviors. Consistent with Shek and Ma's study, linear mixed-effect modeling via SPSS was used to analyze six waves of data collected during three consecutive years.

## METHOD

### Participants and Procedures

The detailed procedure and criteria of recruiting participants for the randomized controlled group trial were described elsewhere[20]. In brief, 24 experimental schools and 24 control schools were randomly selected in Year 1, however, one experimental school dropped out after the first-year implementation of the Project. Therefore, Wave 1 and Wave 2 data were collected from Secondary 1 students in 23 experimental schools and 24 control schools. In Year 2, Wave 3 and Wave 4 data were collected from the same cohort who upgraded to Secondary 2, with 20 experimental schools (i.e., three schools withdrew after Wave 2) and 24 control schools. In Year 3, Wave 5 and Wave 6 data were collected from the same cohort in Secondary 3 at that time, including 19 experimental schools (i.e., one experimental school dropped out after Wave 4) and 24 control schools. Table 1 shows the number of completed questionnaires collected in each wave.

**TABLE 1**
**Number of Collected Questionnaires Across Waves**

| | Wave 1 | Wave 2 | Wave 3 | Wave 4 | Wave 5 | Wave 6 |
|---|---|---|---|---|---|---|
| n (School) | 48 | 47[a] | 44[b] | 44 | 43[c] | 43 |
| No. of participants | 7,846 | 7,388 | 6,939 | 6,697 | 6,876 | 6,733 |
| Control group | 3,797 | 3,654 | 3,765 | 3,698 | 3,757 | 3,727 |
| Male | 1,936 | 1,876 | 1,896 | 1,888 | 1,874 | 1,894 |
| Female | 1,613 | 1,619 | 1,666 | 1,599 | 1,682 | 1,679 |
| Experimental group | 4,049 | 3,734 | 3,174 | 2,999 | 3,119 | 3,006 |
| Male | 2,154 | 1,998 | 1,691 | 1,548 | 1,632 | 1,591 |
| Female | 1,745 | 1,571 | 1,283 | 1,259 | 1,312 | 1,278 |
| % of successfully matched | 98% | 96% | 97% | 98% | 99% | 97% |

a One experimental school (n = 207) had withdrawn after Wave 1.

b Three experimental schools (n = 629) had withdrawn after Wave 2.

c One experimental school (n = 71) had withdrawn after Wave 4.

At each measurement occasion, the purposes of the study were introduced and confidentiality of the data collected was repeatedly ensured to all participants in attendance on the days of survey. Parental and student consent forms was obtained before data collection. Participants responded to the questionnaires in a self-administration format in classroom settings. A trained research assistant was present throughout the administration process.

## Instruments

Consistent with procedures employed in previous studies, participants were required to respond to a questionnaire that included measures of youth development and problem behaviors. The measures in the questionnaire are outlined in the following sections. The internal consistency of each measure on the present sample is presented in Table 2.

**TABLE 2**
**Internal Consistency and Mean Interitem Correlations for Composite Problem Behavior Indicators**

| | Wave 1 | | Wave 2 | | Wave 3 | | Wave 4 | | Wave 5 | | Wave 6 | |
|---|---|---|---|---|---|---|---|---|---|---|---|---|
| | α | Mean[a] | α | Mean[a] | α | Mean[a] | α | Mean[a] | α | Mean[a] | α | Mean[a] |
| DELINQ | 0.77 | 0.32 | 0.79 | 0.35 | 0.79 | 0.35 | 0.82 | 0.40 | 0.81 | 0.38 | 0.82 | 0.38 |
| DRUG | 0.76 | 0.56 | 0.81 | 0.58 | 0.77 | 0.56 | 0.82 | 0.61 | 0.79 | 0.59 | 0.83 | 0.63 |
| BEINT | 0.76 | 0.47 | 0.78 | 0.47 | 0.79 | 0.49 | 0.78 | 0.46 | 0.79 | 0.47 | 0.79 | 0.46 |

*Note*: DELINQ: delinquency; DRUG: substance abuse; BEINT: problem behavior intention.

[a] Mean interitem correlation.

All parameters were significant ($p < 0.05$).

### *Chinese Positive Youth Development Scale (CPYDS)*

The CPYDS consists of 15 subscales, which are listed as follows:

1. Bonding Subscale (six items)
2. Resilience Subscale (six items)
3. Social Competence Subscale (seven items)
4. Emotional Competence Subscale (six items)
5. Cognitive Competence Subscale (six items)
6. Behavioral Competence Subscale (modified five items)
7. Moral Competence Subscale (six items)
8. Self-Determination Subscale (five items)
9. Self-Efficacy Subscale (modified two items)
10. Beliefs in the Future Subscale (modified three items)
11. Clear and Positive Identity Subscale (seven items)
12. Spirituality Subscale (seven items)
13. Prosocial Involvement Subscale (five items)
14. Prosocial Norms Subscale (five items)
15. Recognition for Positive Behavior Subscale (four items)

It should be noted that although the administered questionnaire includes the CPYDQ, findings based on CPYDQ and its subscales were reported elsewhere[21]. The present paper focuses only on the development of problem behaviors among the participants, including delinquent behavior, substance abuse, intention of engaging in problem behavior in the future, and Internet use control, as measured by the following scales.

### *Delinquency Scale*

This scale comprises 12 items that assess the frequency of delinquent behavior of the participants in the past half year, including stealing, cheating, truancy, running away from home, damaging others' properties, assault, having sexual intercourse with others, gang fighting, speaking foul language, staying outside the home overnight without parental consent, strong arming others, and trespasses[22]. Respondents rated the frequency of these behaviors in the past half year on a six-point Likert scale (0 = never, 1 = one to two times; 2 = three to four times; 3 = five to six times; 4 = seven to eight times; 5 = nine to 10 times; 6 = more than 10 times).

### *Substance Abuse Scale*

Eight items were used to assess the participants' frequency of using different types of substances in the past half year, including alcohol, tobacco, ketamine, cannabis, cough mixture, organic solvent, pills (including ecstasy and methaqualone), and heroin. Participants rated their occurrence of these behaviors on a six-point Likert scale (0 = never; 1 = one to two times; 2 = three to five times; 3 = more than five times; 4 = several times a month; 5 = several times a week; 6 = every day). Because the severity and meaning of consuming different substances (e.g., alcohol and heroin) is not the same, separate analyses were carried out for different types of drugs. In addition, a composite score of illegal psychotropic drug use was calculated by averaging the item scores on ketamine use, cannabis use, taking pills, and heroin use, considering their similar developmental trends among adolescents.

### *Problem Behavior Intention Scale*

Five items were used to assess the participants' behavioral intention to engage in problem behavior, including drinking alcohol, smoking, taking drugs (such as ketamine, cannabis, or ecstasy), having sex with others, and gambling[23]. Respondents were asked to rate the likelihood that they may engage in these problem behaviors in the next 2 years on a four-point Likert scale, with "1" representing "never", "2" for "not likely", "3" for "likely", and "4" for "definitely".

### *Internet Use Control*

Given that Internet addiction has become an important adolescent health issue, the present study also used one item to assess students' self-perceived ability to control their Internet use. Participants were asked to report the extent to which they would agree with the statement that "I am able to control my use of Internet" on a six-point Likert scale (1 = strongly disagree; 2 = disagree; 3 = slightly disagree; 4 = slightly agree; 5 = agree; 6 = strongly agree), with high scores representing high self-control over Internet use.

## Data Analytic Plan

In the present study, we adopted the individual growth curve (IGC) modeling approach, as recommended by Shek and Ma[19], to analyze adolescents' individual change in problem behaviors over time and to examine the longitudinal effects of the Project P.A.T.H.S. on the developmental trajectories of different youth problem behaviors. Both composite indicators (i.e., scale scores of delinquency, substance abuse, and problem behavior intention, and the composite score of illegal psychotropic drug use) and individual item scores were treated as dependent variables.

The use of IGC in studying longitudinal data has been detailed in many articles[24]. In a nutshell, longitudinal data are considered as a two-level hierarchical model in which time is nested within individuals[25,26]. The Level 1 model refers to the intraindividual change model that models the variation within an individual over time, and estimates the average within-person initial status and the average rate of change over time. In other words, the outcome variable is represented as simply the function of time without any other predictors involved. The Level 2 model captures whether the rate of change varies across individuals in a systematic way. The growth parameters estimated in the Level 1 model serve as the outcome variables in the Level 2 model, which are further predicted by various interindividual variables. At this step, different explanatory variables such as "participation in the program" (i.e., experimental group vs. control group) can be included to analyze their effects on the interindividual variation of outcome variables.

More information about how to formulate and interpret the IGC models can be seen in the paper by Shek and Ma[24]. To iterate, the longitudinal effects of the program on youth problem behavior were tested by examining whether "participating in the Project P.A.T.H.S." was predictive of students' growth parameters (i.e., initial status, linear change, quadratic change, and cubic change) in different problem behavior indicators across time, with the effects of gender and initial age being controlled. In the IGC models, the intercept (i.e., initial status) and linear slope were allowed to vary across individuals.

First, a dummy/dichotomous variable was created (i.e., *group* — experimental group vs. control group) as a major predictor. Participants in the control group were coded as -1 and those in the experimental group as 1. Two covariates (i.e., gender and initial age) were included when examining the effects of predictive program on the outcome variables. *Gender* was coded as -1 = male and 1 = female. Following Shek and Ma's method[19], continuous variables were grand-mean centered in order to simplify the interpretation of the results[21]. In this study, the mean age was 12. *Initial age* was then centered by subtracting the mean age and, therefore, the centered initial age was generated.

To facilitate the interpretation of the significant interaction effects (between time variables and the program), prototypical trajectories were plotted as suggested by Singer and Willett[27] to illustrate the effect of treatment on the rate of change across time. The step in creating prototypical plots is generally identical to the method of plotting graphs in regression[28]. For each outcome variable, a Linear Mixed Model (LMM) via SPSS with maximum likelihood estimation was conducted. Because the focus was on the entire model (both fixed and random effects), the Maximum Likelihood (ML) method was used[29]. The procedures for analyzing longitudinal data via SPSS can be seen in Shek and Ma[24].

## RESULTS

With schools as the units of analysis, results indicated that the 19 experimental schools and 24 control schools did not differ in school characteristics in terms of banding (i.e., categorizing based on students academic competence), geographic district, religious affiliation, sex ratio of the students, and source of funding. At the individual level, preliminary analyses showed that there were no statistically significant differences between the two groups in all sociodemographic background characteristics of the students ($p > 0.05$), except age. The mean age of the control group was higher than that of the experimental group. In other words, the background characteristics of the experimental schools and control schools were highly comparable at Wave 1.

Table 3 presents the IGC findings based on different problem behavior indicators. Results show that the effects of group were nonsignificant on any slope of the three scale scores: delinquency, substance abuse, and problem behavior intention. Using individual item score as the dependent variable, significant interaction of group and slopes were found in four substance abuse behaviors ( use of ketamine, cannabis, pills, and heroin) and four delinquent behaviors (having sexual experience, running away from home, staying outside the home overnight without parental approval, and trespasses). In addition, significant group effect was found in the development of the composite indicator of illegal psychotropic drug use.

**TABLE 3**
**Growth Curve Models for Problem Behavior Indicators with Subjects who Joined the Tier 1 Program as Experimental Subjects**

| | Dependent Variables | | | | | | | | | | | |
|---|---|---|---|---|---|---|---|---|---|---|---|---|
| | **DELIN** | **DRUG** | **BEINT** | **KETA** | **CANNA** | **PILL** | **HERN** | **PSYTR** | **RUN** | **SEX** | **STOH** | **TRESP** |
| Intercept | | | | | | | | | | | | |
| Initial status | 0.27** | 0.08** | 10.23** | 0.00 | 0.01 | 0.01 | 0.00 | 0.01 | 0.06** | 0.02** | 0.09** | 0.05** |
| Group | -0.03** | -0.01 | -0.02* | 0.00 | 0.00 | 0.00 | 0.00 | 0.00 | -0.02** | 0.00 | -0.01 | -0.01 |
| Gender | -0.11** | -0.03** | -0.08** | 0.00 | 0.00 | 0.00 | 0.00 | 0.00 | -0.04** | -0.03* | -0.06** | -0.02 |
| Age | 0.06** | 0.04** | 0.06** | 0.01* | 0.01 | 0.01* | 0.01 | 0.01* | 0.04** | 0.02** | 0.09** | 0.02** |
| Linear | | | | | | | | | | | | |
| Initial status | 0.25** | 0.06* | 0.20** | 0.02** | 0.01** | 0.01** | 0.01** | 0.01** | 0.03** | 0.03** | 0.05** | 0.02** |
| Group | -0.03 | -0.01 | 0.00 | -0.01* | -0.004[a] | -0.003[a] | -0.004[a] | -0.004* | -0.01* | -0.01* | -0.02** | -0.01* |
| Gender | 0.07 | 0.03 | 0.07 | -0.01** | -0.01** | -0.01** | -0.01** | -0.01** | -0.01 | 0.00 | -0.02* | -0.02* |
| Age | -0.02 | 0.01 | 0.02 | 0.00 | -0.01** | 0.00 | -0.005* | -0.004* | -0.01 | 0.00 | -0.01 | -0.01* |
| Quadratic | | | | | | | | | | | | |
| Initial status | -0.06* | 0.00 | -0.04 | — | — | — | — | — | — | — | — | — |
| Group | 0.02 | 0.00 | 0.00 | — | — | — | — | — | — | — | — | — |
| Gender | -0.10** | -0.02 | -0.07 | — | — | — | — | — | — | — | — | — |
| Age | -0.01 | -0.02 | -0.03 | — | — | — | — | — | — | — | — | — |
| Cubic | | | | | | | | | | | | |
| Initial status | 0.01 | 0.00 | 0.01 | — | — | — | — | — | — | — | — | — |
| Group | 0.00 | 0.00 | 0.00 | — | — | — | — | — | — | — | — | — |
| Gender | 0.02** | 0.00 | 0.01 | — | — | — | — | — | — | — | — | — |
| Age | 0.00 | 0.00 | 0.01 | — | — | — | — | — | — | — | — | — |

*Note*: DELIN = scale score of the Delinquency Scale; DRUG = scale score of the Substance Abuse Scale; BEINT = scale score of the Intention of Problem Behavior Scale; KETA = item score of using ketamine; CANNA = item score of using cannabis; PILL = item score of taking pills (e.g., ecstasy or methaqualone); HERN = item score of using heroin; PSYTR = composite score of illegal psychotropic drug use; RUN = item score of running away from home; SEX = item score of having sexual intercourse; STOH = item score of staying outside the home overnight without parental approval; TRESP = item score of trespasses.
$p < 0.10$[a], $p < 0.05$*, $p < 0.01$** (all $p$ values shown in the table were based on two-tailed tests).

Ketamine use — For both the experimental group and the control group, participants' use of ketamine increased over time, following a linear developmental trend. Group was a significant predictor of the linear slope, but was unrelated to the initial status ($p > 0.05$). While the nonsignificant group effect on the initial status suggested that the two groups did not differ in ketamine use at the beginning of the study, group difference in the linear slope ($\beta$ = -0.01, $SE$ = 0.002, $p < 0.05$) indicated that the use of ketamine increased faster in the control group than in the experimental group. As can be seen in Fig. 1, the discrepancy in ketamine use between the two groups became larger when time increased.

Cannabis use — Similar to ketamine use, cannabis use in the participants also followed a linear trajectory, with participants' use of cannabis increasing with time. Group had a significant effect on the linear slope ($\beta$ = -0.004, $SE$ = 0.002, $p$ = 0.04, one-tailed test), meaning that the increase of cannabis use in the experimental group was slower than that in the control group (Fig. 2).

Taking pills — Participants' behavior of taking pills, such as ecstasy or methaqualone, increased linearly over time. Group significantly predicted the linear slope ($\beta$ = -0.004, $SE$ = 0.002, $p$ = 0.045, one-tailed test). As can be seen in Fig. 3, participants' use of ecstasy or methaqualone increased faster in the control group than in the experimental group.

Heroin use — As with the other three types of substance use, heroin use also showed a linear increase over time and group interacted with the linear slope significantly ($\beta$ = -0.004, $SE$ = 0.002, $p$ = 0.04, one-tailed test). Again, the increase of heroin use with time in the control participants was faster than that in the experimental participants (Fig. 4).

**Ketamine**

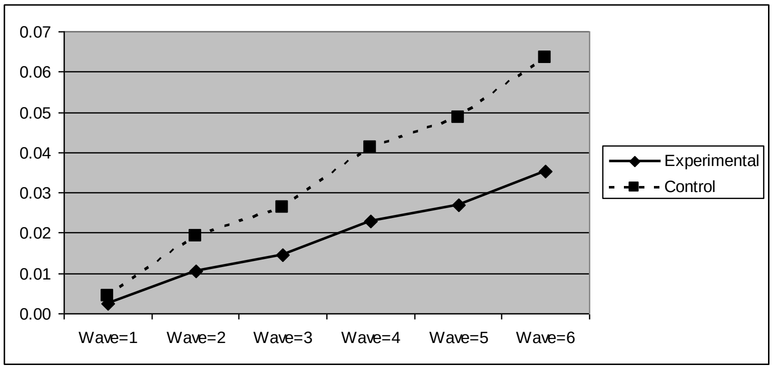

**FIGURE 1**. Growth trajectories of the experimental group (participants who joined the Tier 1 Program only) and control group using the item score of ketamine use as the outcome indicator.

**Cannabis**

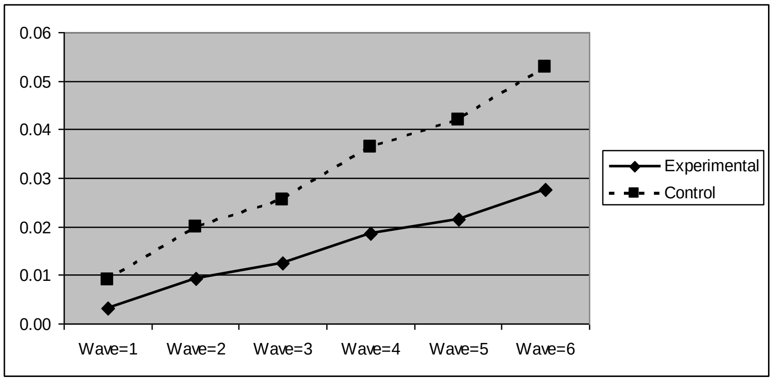

**FIGURE 2**. Growth trajectories of the experimental group (participants who joined the Tier 1 Program only) and control group using the item score of cannabis use as the outcome indicator.

**Pills**

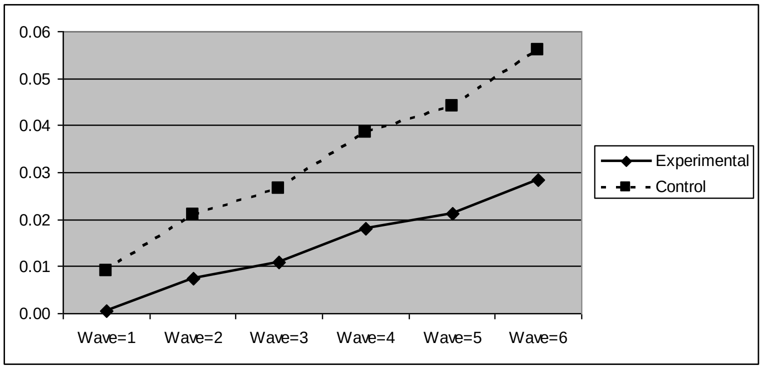

**FIGURE 3**. Growth trajectories of the experimental group (participants who joined the Tier 1 Program only) and control group using the item score of taking pills as the outcome indicator.

Psychotropic drug use — Using the composite score of use of four illegal psychotropic drugs as the indicator, IGC results showed that group significantly predicted its linear slope ($\beta$ = -0.004, $SE$ = 0.002, $p < 0.05$), but was unrelated to the initial status ($p > 0.05$). This means that although the use of illegal psychotropic drugs did not differ between the two groups at the beginning of the study, the increase of these drug use behaviors in the control group was faster compared to the experimental group (Fig. 5).

**Heroin**

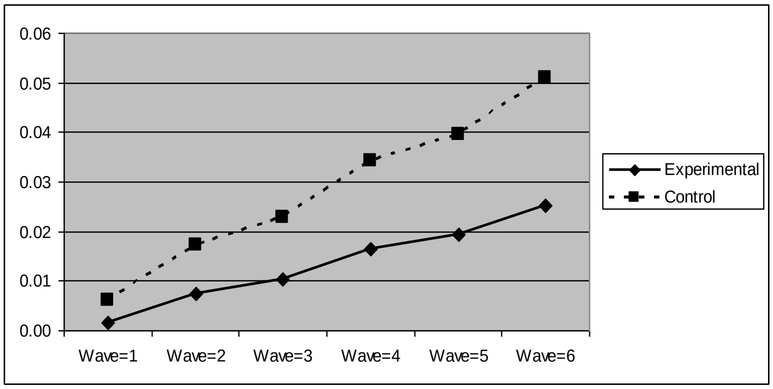

**FIGURE 4**. Growth trajectories of the experimental group (participants who joined the Tier 1 Program only) and control group using the item score of heroin use as the outcome indicator.

**Psychotropic Drugs**

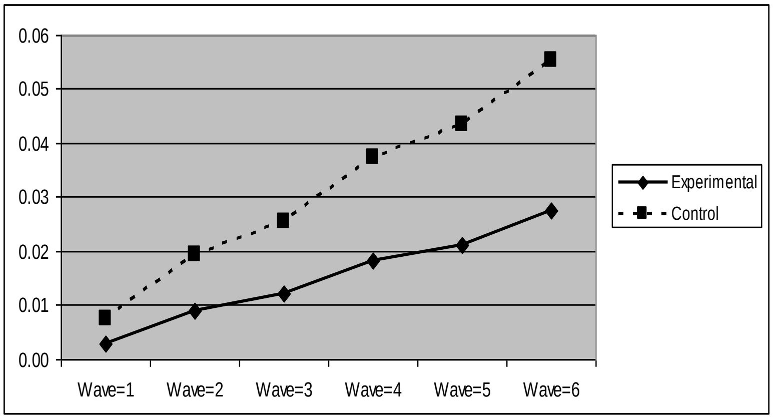

**FIGURE 5**. Growth trajectories of the experimental group (participants who joined the Tier 1 Program only) and control group using the composite score of psychotropic drug use as the outcome indicator.

Delinquent behaviors — The four delinquent behaviors showing significant group and slope interactions exhibited similar linear developmental trajectories, with participants' delinquent behaviors increasing with time. The effects of group on the developmental trends of these behaviors also displayed similar patterns. Group was negatively related to the linear slopes of the four delinquent behaviors. Specifically, for "running away from home", $\beta$ = -0.01, *SE* = 0.003, $p < 0.05$; for "having sexual experience", $\beta$ = -0.01, *SE* = 0.003, $p < 0.05$; for "staying outside the home overnight without parental approval", $\beta$ = -0.02, *SE* = 0.01, $p < 0.01$; and for "trespasses", $\beta$ = -0.01, *SE* = 0.003, $p < 0.05$. These results suggested that the increase of these delinquent behaviors in the experimental group was slower than that in the control group. In other words, the program appeared to slow down the deteriorating process of adolescent delinquent behaviors. The developmental curves of the four delinquent behaviors for the experimental group and the control group were plotted in Figs. 6–9.

To provide further support for the effectiveness of the program, participants in the experimental group who perceived the program as beneficial to their development were selected and compared to the control participants. Significant group effects on growth parameters were found in six substance abuse behaviors (smoking, drinking alcohol, ketamine use, cannabis use, taking pills, and heroin use), four delinquent behaviors (damaging other's properties, speaking foul language, staying outside the home overnight without parental approval, and trespasses), and the composite score of illegal psychotropic drug use, as summarized in Table 4. The patterns of group effects on changes in these behaviors were basically consistent with the patterns found previously in comparing all Tier 1 Program participants in the experimental group and the control participants. Growth trajectories of these problem behaviors in the two groups are shown in Figs. 10–20.

**Run Away**

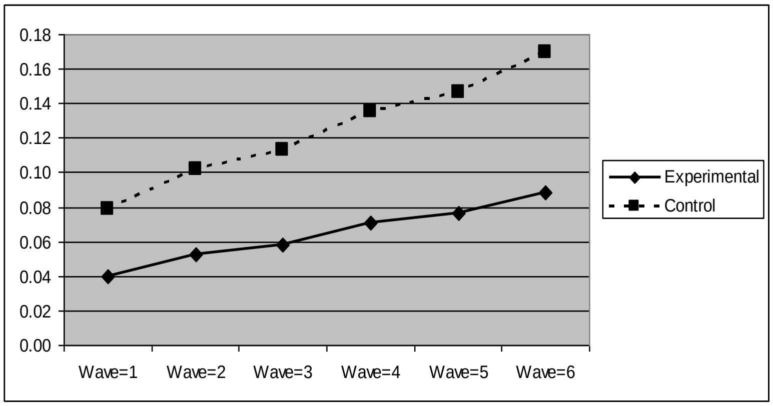

**FIGURE 6**. Growth trajectories of the experimental group (participants who joined the Tier 1 Program only) and control group using the item score of run away from home as the outcome indicator.

**Sex**

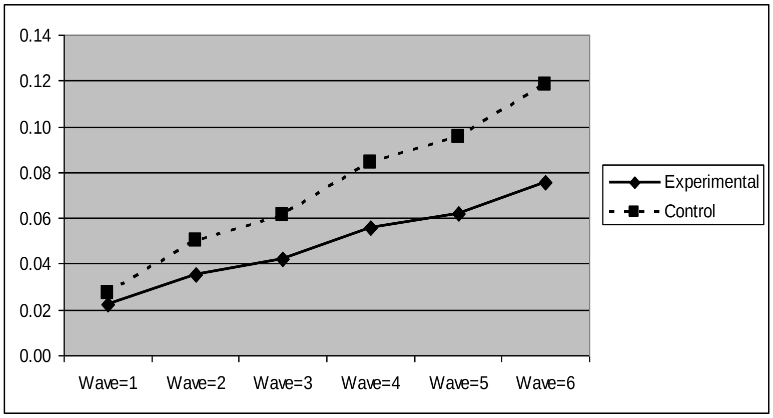

**FIGURE 7**. Growth trajectories of the experimental group (participants who joined the Tier 1 Program only) and control group using the item score of having sexual intercourse as the outcome indicator.

**Staying Outside**

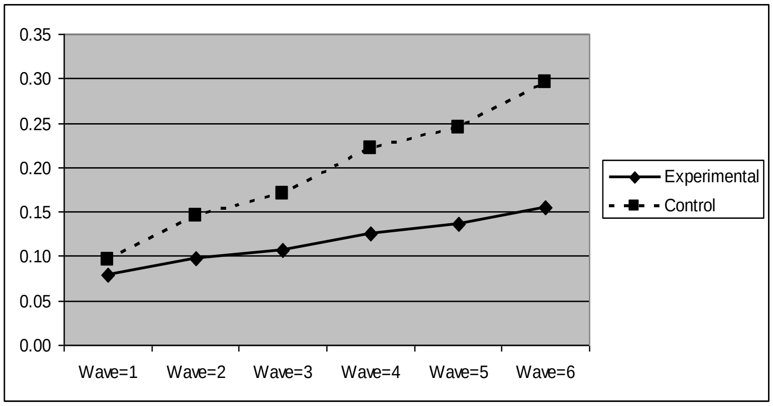

**FIGURE 8**. Growth trajectories of the experimental group (participants who joined the Tier 1 Program only) and control group using the item score of staying outside as the outcome indicator.

**Trespasses**

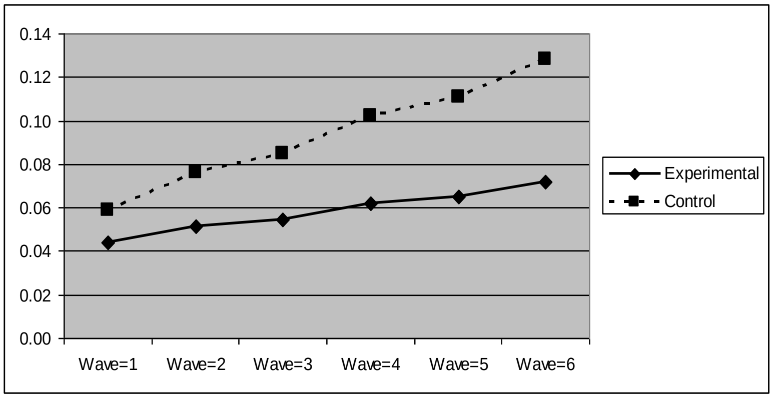

**FIGURE 9**. Growth trajectories of the experimental group (participants who joined the Tier 1 Program only) and control group using the item score of trespasses as the outcome indicator.

**TABLE 4**
**Growth Curve Models for Problem Behavior Indicators with Subjects who Joined the Tier 1 Program and Perceive the Program as Effective being Experimental Subjects**

| | Dependent Variables | | | | | | | | | | | | | | |
|---|---|---|---|---|---|---|---|---|---|---|---|---|---|---|---|
| | **DELIN** | **DRUG** | **BEINT** | **SMOK** | **ALCO** | **KETA** | **CANNA** | **PILL** | **HERN** | **PSYTR** | **NET** | **DAMAG** | **FOUL** | **STOH** | **TRESP** |
| Intercept | | | | | | | | | | | | | | | |
| Initial status | 0.27** | 0.08** | 10.24** | 0.13** | 0.47** | 0.01 | 0.01* | 0.01* | 0.01 | 0.01* | 40.12** | 0.17** | 10.36** | 0.09** | 0.05** |
| Group | -0.03** | -0.01* | -0.01 | -0.02 | -0.03* | 0.00 | 0.00 | 0.00 | 0.00 | 0.00 | 0.07** | -0.01 | -0.15** | -0.01 | 0.00 |
| Gender | -0.11** | -0.03** | -0.10** | -0.06** | -0.15** | 0.00 | -0.01 | 0.00 | -0.01 | -0.01 | 0.39** | -0.10* | -0.35** | -0.05** | -0.01 |
| Age | 0.06** | 0.04** | 0.06** | 0.15** | 0.12** | 0.01** | 0.01** | 0.01** | 0.01 | 0.01** | -0.05* | 0.04 | 0.25** | 0.09** | 0.02** |
| Linear | | | | | | | | | | | | | | | |
| Initial status | 0.31** | 0.09** | 0.21** | 0.16** | 0.28** | 0.02** | 0.01** | 0.01** | 0.01** | 0.01** | -0.26** | 0.12** | 10.43** | 0.06** | 0.02** |
| Group | 0.01 | 0.03 | 0.02 | 0.03 | 0.03 | -0.004[a] | -0.003[a] | -0.004* | -0.004* | -0.004* | 0.13** | -0.03* | 0.11 | -0.01* | -0.01* |
| Gender | 0.03 | 0.02 | 0.07* | 0.06* | 0.15** | -0.01* | -0.01** | -0.01** | -0.01** | -0.01** | -0.48** | -0.04 | 0.35* | -0.03* | -0.02** |
| Age | -0.02 | 0.02 | 0.03 | 0.04* | 0.00 | 0.00 | 0.00 | 0.00 | 0.00 | 0.00 | 0.09 | 0.00 | -0.27** | 0.00 | -0.01* |
| Quadratic | | | | | | | | | | | | | | | |
| Initial status | -0.13** | -0.03 | -0.06* | -0.01 | -0.03 | — | | | — | — | 0.10 | -0.02* | -0.56** | — | — |
| Group | -0.03 | -0.03* | -0.01 | -0.01* | -0.01[a] | — | | | — | — | -0.13** | 0.01* | -0.15* | — | — |
| Gender | -0.07 | -0.01 | -0.07* | -0.03** | -0.05** | — | | | — | — | 0.33** | 0.00 | -0.37** | — | — |
| Age | 0.00 | -0.01 | -0.03 | -0.01* | 0.00 | — | | | — | — | -0.05 | 0.00 | 0.12 | — | — |
| Cubic | | | | | | | | | | | | | | | |
| Initial status | 0.02** | 0.01 | 0.01 | — | — | — | | | — | — | -0.01 | — | 0.09** | — | — |
| Group | 0.01* | 0.01* | 0.00 | — | — | — | | | — | — | 0.03** | — | 0.03 | — | — |
| Gender | 0.01 | 0.00 | 0.01 | — | — | — | | | — | — | -0.07** | — | 0.07* | — | — |
| Age | 0.00 | 0.00 | 0.01 | — | — | — | | | — | — | 0.01 | — | -0.02 | — | — |

*Note*: DELIN = scale score of the Delinquency Scale; DRUG = scale score of the Substance Abuse Scale; BEINT = scale score of the Intention of Problem Behavior Scale; SMOK = item score of smoking behavior; ALCO = item score of drinking alcohol; KETA = item score of using ketamine; CANNA = item score of using cannabis; PILL = item score of taking pills (e.g., ecstasy or methaqualone); HERN = item score of using heroin; PSYTR = composite score of illegal psychotropic drug use; NET = item score of ability to control Internet use; DAMAG = item score of destroying other's properties; FOUL = item score of speaking foul language; STOH = item score of staying outside the home overnight without parental approval; TRESP = item score of trespasses.

$p < 0.10^a$, $p < 0.05^*$, $p < 0.01^{**}$ (all $p$ values shown in the table were based on two-tailed tests).

Moreover, another behavioral indicator, students' ability to control Internet use, with a cubic developmental trend, showed significant group differences in its growth parameters. Group significantly predicted the initial status ($\beta = 0.07$, $SE = 0.02$, $p < 0.01$), linear slope ($\beta = 0.13$, $SE = 0.05$, $p < 0.01$), quadratic slope ($\beta = -0.13$, $SE = 0.05$, $p < 0.01$), and cubic slope ($\beta = 0.03$, $SE = 0.01$, $p < 0.01$). As can be seen in Fig. 21, students' ability to control Internet use in the control group decreased more quickly followed by a faster deceleration than the experimental group. More interestingly, the interactive effect between group and cubic slope suggested that while the experimental participants' control over Internet use showed a tendency toward increase at later waves, such ability in the control participants continued to deteriorate.

**Smoking**

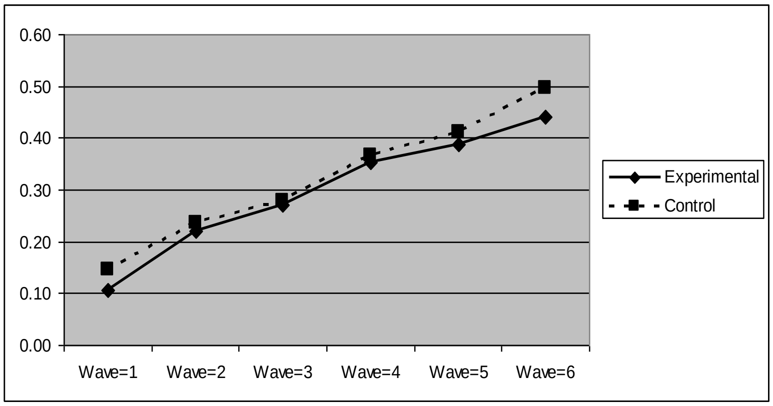

**FIGURE 10**. Growth trajectories of the experimental group (Tier 1 participants who perceived the program as effective) and control group using the item score of smoking as the outcome indicator.

**Alcohol**

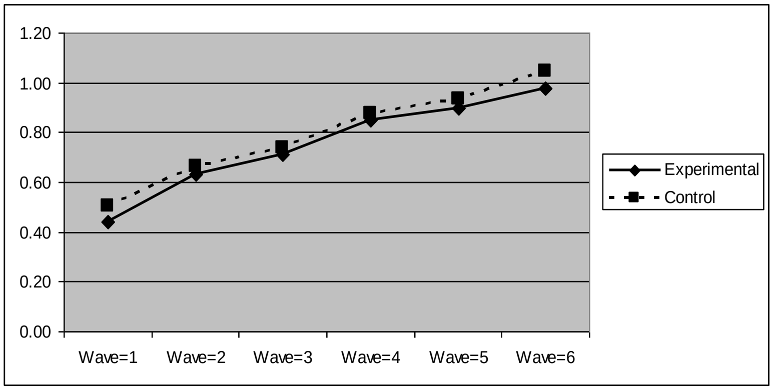

**FIGURE 11**. Growth trajectories of the experimental group (Tier 1 participants who perceived the program as effective) and control group using the item score of drinking alcohol as the outcome indicator.

**Ketamine**

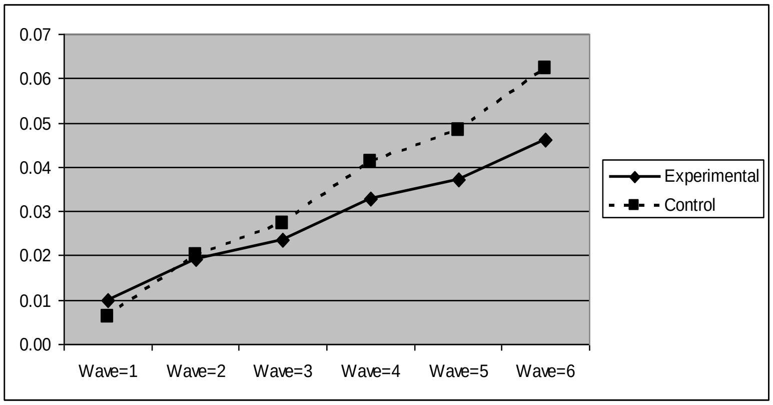

**FIGURE 12**. Growth trajectories of the experimental group (Tier 1 participants who perceived the program as effective) and control group using the item score of ketamine use as the outcome indicator.

**Cannabis**

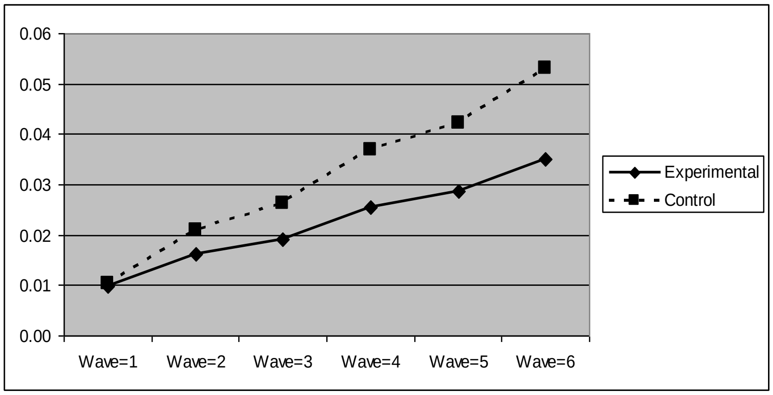

**FIGURE 13**. Growth trajectories of the experimental group (Tier 1 participants who perceived the program as effective) and control group using the item score of cannabis use as the outcome indicator.

**Pills**

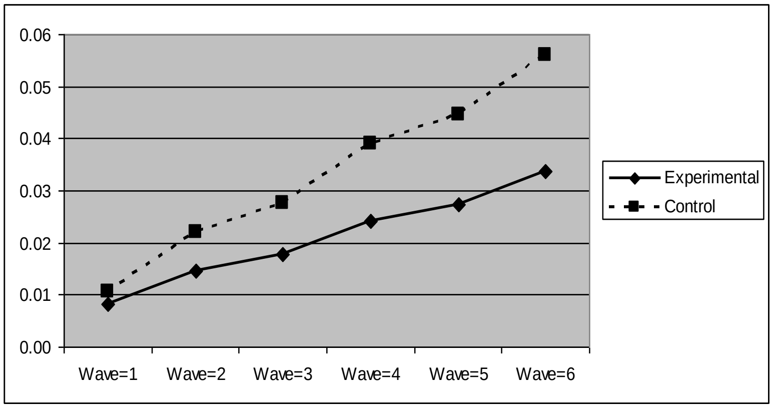

**FIGURE 14**. Growth trajectories of the experimental group (Tier 1 participants who perceived the program as effective) and control group using the item score of taking pills as the outcome indicator.

**Heroin**

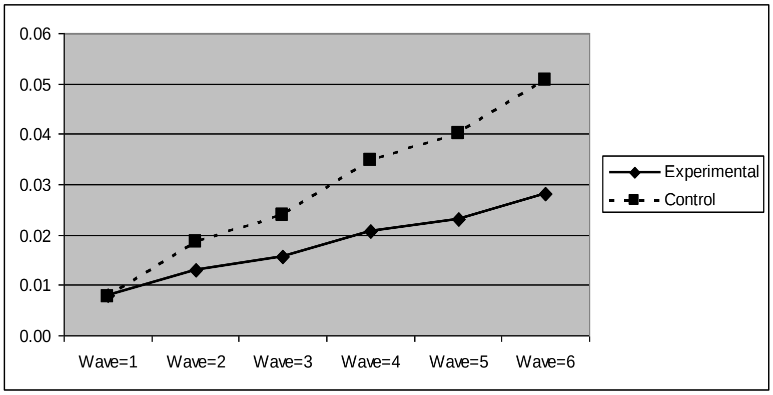

**FIGURE 15**. Growth trajectories of the experimental group (Tier 1 participants who perceived the program as effective) and control group using the item score of heroin use as the outcome indicator.

**Psychotropic Drugs**

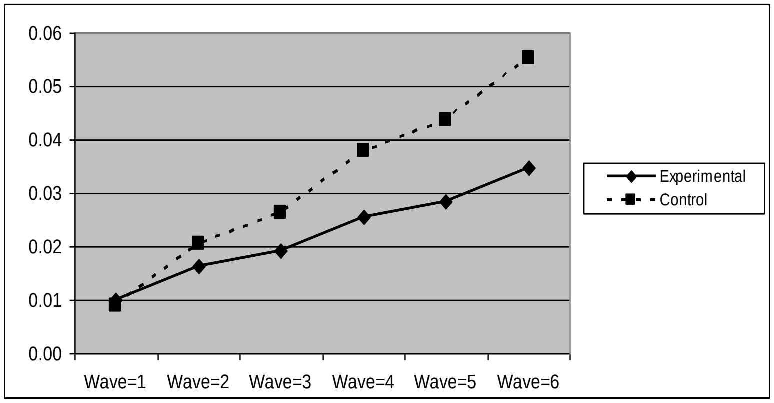

**FIGURE 16**. Growth trajectories of the experimental group (Tier 1 participants who perceived the program as effective) and control group using the composite score of psychotropic drug use as the outcome indicator.

**Damage**

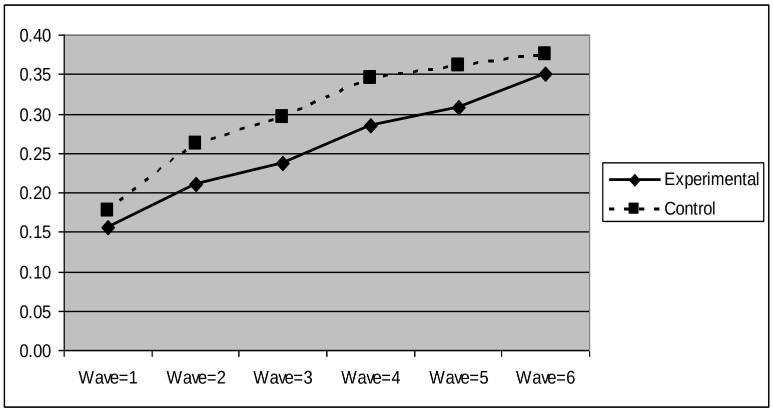

**FIGURE 17**. Growth trajectories of the experimental group (Tier 1 participants who perceived the program as effective) and control group using the item score of damaging other's properties as the outcome indicator.

**Foul Language**

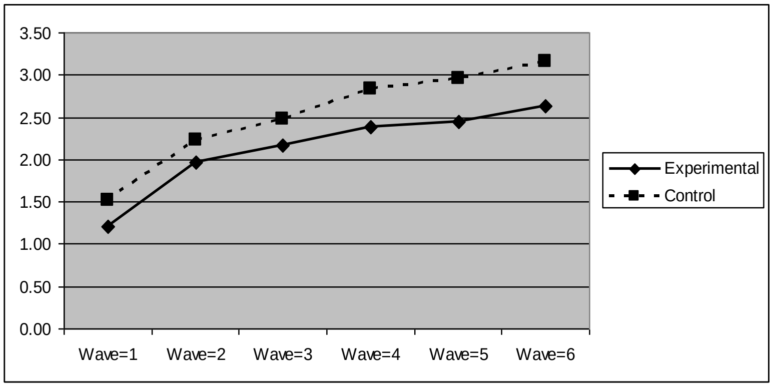

**FIGURE 18**. Growth trajectories of the experimental group (Tier 1 participants who perceived the program as effective) and control group using the item score of speaking foul language as the outcome indicator.

**Staying Outside**

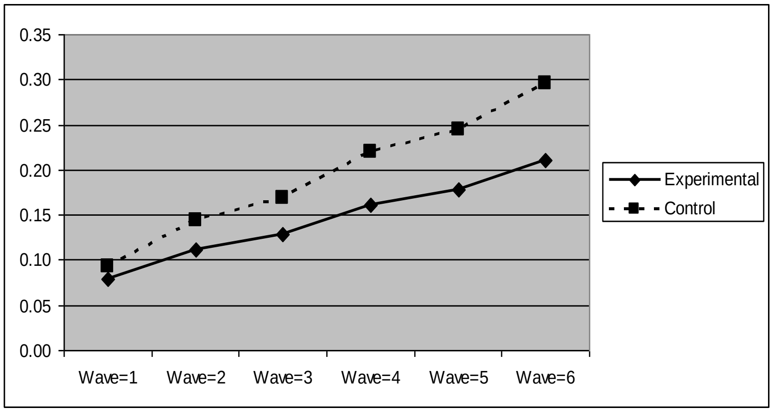

**FIGURE 19**. Growth trajectories of the experimental group (Tier 1 participants who perceived the program as effective) and control group using the item score of staying outside as the outcome indicator.

**Trespasses**

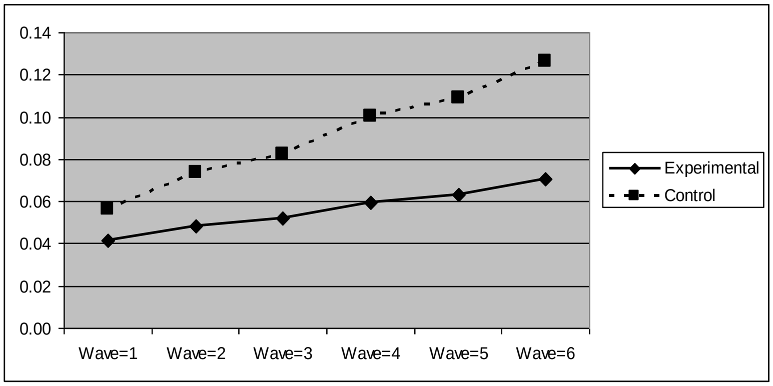

**FIGURE 20**. Growth trajectories of the experimental group (Tier 1 participants who perceived the program as effective) and control group using the item score of trespasses as the outcome indicator.

**Internet Use Control**

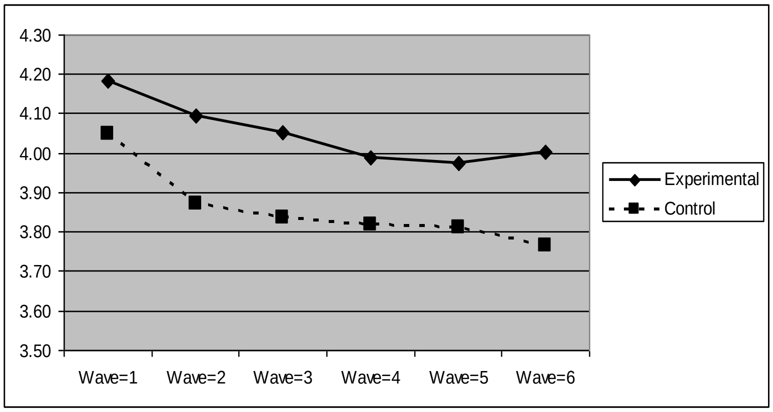

**FIGURE 21**. Growth trajectories of the experimental group (Tier 1 participants who perceived the program as effective) and control group using the item score of ability to control the Internet as the outcome indicator.

To further evaluate the effect size of group in explaining the between-individual variation in the growth curves, the Pseudo-$R^2$ was calculated for each behavioral indicator with significant time and group interaction effects. The results are summarized in Table 5 (comparing control group and Tier 1 Program participants) and Table 6 (comparing control group and participants who perceived the Tier 1 Program as effective). In the tables, M0 refers to the baseline model, M1 refers to the model with only the

**TABLE 5**
**Pseudo-$R^2$ for IGC with Subjects who Joined the Tier 1 Program being Experimental Subjects**

| | | Residual (Within Individual) | Intercept Variance | Time Variance | Intercept-Time Variance |
|---|---|---|---|---|---|
| KETA | Compare M1 and M0 | 0.24 | 0.39 | 0.44 | -0.27 |
| | Compare M2 and M0 | 0.32 | 0.71 | 0.51 | 0.64 |
| | Compare M3 and M0 | 0.37 | 1 | 0.61 | 0.73 |
| | Compare M3 and M2 | 0.06 | 1 | 0.20 | 0.26 |
| CANNA | Compare M1 and M0 | 0.20 | 0.40 | 0.13 | 0.27 |
| | Compare M2 and M0 | 0.28 | 0.73 | 0.64 | 0.69 |
| | Compare M3 and M0 | 0.27 | 1 | 0.71 | -9.78 |
| | Compare M3 and M2 | -0.02 | 1 | 0.19 | -33.62 |
| PILL | Compare M1 and M0 | 0.18 | 0.48 | 0.04 | 0.28 |
| | Compare M2 and M0 | 0.32 | 0.67 | 0.51 | 0.60 |
| | Compare M3 and M0 | 0.30 | 1 | 0.63 | -11.51 |
| | Compare M3 and M2 | -0.02 | 1 | 0.24 | -30.33 |
| HERN | Compare M1 and M0 | 0.09 | 1 | 0.32 | -0.04 |
| | Compare M2 and M0 | 0.28 | 0.80 | 0.60 | 0.71 |
| | Compare M3 and M0 | 0.32 | 1 | 0.71 | 0.94 |
| | Compare M3 and M2 | 0.04 | 1 | 0.28 | 0.80 |
| PSYTR | Compare M1 and M0 | 0.22 | 0.50 | 0.07 | 0.32 |
| | Compare M2 and M0 | 0.30 | 0.70 | 0.55 | 0.63 |
| | Compare M3 and M0 | 0.33 | 1 | 0.67 | -8.64 |
| | Compare M3 and M2 | 0.03 | 1 | 0.26 | -25.19 |
| RUN | Compare M1 and M0 | 0.10 | 0.07 | 0.44 | 0.42 |
| | Compare M2 and M0 | 0.17 | 0.49 | 0.14 | 0.66 |
| | Compare M3 and M0 | 0.20 | 0.49 | 0.44 | 0.78 |
| | Compare M3 and M2 | 0.04 | -0.01 | 0.35 | 0.41 |
| SEX | Compare M1 and M0 | 0.13 | - | 0.23 | 0.30 |
| | Compare M2 and M0 | 0.22 | - | -12.61 | 0.98 |
| | Compare M3 and M0 | 0.35 | - | 0.44 | 0.88 |
| | Compare M3 and M2 | 0.13 | - | 0.96 | -3.78 |
| STOH | Compare M1 and M0 | 0.10 | 0.10 | 0.11 | 0.46 |
| | Compare M2 and M0 | 0.18 | 0.16 | 0.19 | 0.62 |
| | Compare M3 and M0 | 0.25 | 0.24 | 0.18 | 0.83 |
| | Compare M3 and M2 | 0.07 | 0.10 | -0.02 | 0.55 |
| TRESP | Compare M1 and M0 | 0.06 | 0.57 | -0.47 | 0.21 |
| | Compare M2 and M0 | 0.14 | 0.46 | -0.03 | 0.25 |
| | Compare M3 and M0 | 0.20 | 0.77 | -0.10 | 0.32 |
| | Compare M3 and M2 | 0.06 | 0.58 | -0.94 | 0.10 |

*Note:* M0 = Baseline model; M1 = Model 1, include predictor (group) with no covariates; M2 = Model 2, include two covariates (initial age and gender); M3 = Model 3, include predictor (group) and two covariates (initial age and gender). KETA = item score of using ketamine; CANNA = item score of using cannabis; PILL = item score of taking pills (e.g., ecstasy or methaqualone); HERN = item score of using heroin; PSYTR = composite score of illegal psychotropic drug use; RUN = item score of running away from home; SEX = item score of having sexual intercourse; STOH = item score of staying outside the home overnight without parental approval; TRESP = item score of trespasses.

**TABLE 6**
**Pseudo-$R^2$ for IGC with Subjects who Joined the Tier 1 Program and Perceived the Program as Effective being Experimental Subjects**

| | | Residual (Within Individual) | Intercept Variance | Time Variance | Intercept-Time Variance |
|---|---|---|---|---|---|
| DELIN | Compare M1 and M0 | 0.08 | 0.10 | 0.17 | 0.13 |
| | Compare M2 and M0 | 0.09 | 0.25 | 0.20 | 0.30 |
| | Compare M3 and M0 | 0.16 | 0.33 | 0.29 | 0.36 |
| | Compare M3 and M2 | 0.06 | 0.11 | 0.11 | 0.08 |
| DRUG | Compare M1 and M0 | 0.18 | 0.13 | 0.26 | 0.19 |
| | Compare M2 and M0 | 0.12 | 0.60 | 0.46 | 0.36 |
| | Compare M3 and M0 | 0.25 | 0.72 | 0.52 | 0.45 |
| | Compare M3 and M2 | 0.12 | 0.30 | 0.10 | 0.14 |
| SMOK | Compare M1 and M0 | 0.08 | 0.13 | 0.15 | 0.04 |
| | Compare M2 and M0 | 0.14 | 0.24 | 0.19 | 0.24 |
| | Compare M3 and M0 | 0.19 | 0.39 | 0.27 | 0.19 |
| | Compare M3 and M2 | 0.05 | 0.19 | 0.11 | -0.07 |
| ALCO | Compare M1 and M0 | 0.06 | 0.02 | 0.05 | 0.12 |
| | Compare M2 and M0 | 0.06 | 0.14 | 0.07 | 0.00 |
| | Compare M3 and M0 | 0.10 | 0.17 | 0.10 | -0.02 |
| | Compare M3 and M2 | 0.04 | 0.03 | 0.03 | -0.03 |
| KETA | Compare M1 and M0 | 0.21 | 0.32 | 0.12 | 0.23 |
| | Compare M2 and M0 | 0.31 | 0.71 | 0.52 | 0.63 |
| | Compare M3 and M0 | 0.30 | 1 | 0.61 | -12.03 |
| | Compare M3 and M2 | -0.01 | 1 | 0.20 | -34.06 |
| CANNA | Compare M1 and M0 | 0.16 | 0.20 | 0.30 | 0.25 |
| | Compare M2 and M0 | 0.28 | 0.79 | 0.68 | 0.73 |
| | Compare M3 and M0 | 0.38 | 0.90 | 0.77 | 0.85 |
| | Compare M3 and M2 | 0.10 | 0.49 | 0.30 | 0.42 |
| PILL | Compare M1 and M0 | 0.19 | 0.24 | 0.27 | 0.26 |
| | Compare M2 and M0 | 0.33 | 0.68 | 0.50 | 0.60 |
| | Compare M3 and M0 | 0.27 | 1 | 0.68 | 0.52 |
| | Compare M3 and M2 | -0.05 | 1 | 0.35 | -0.21 |
| HERN | Compare M1 and M0 | 1 | 1 | 1 | 1 |
| | Compare M2 and M0 | 0.28 | 0.80 | 0.60 | 0.71 |
| | Compare M3 and M0 | 0.24 | 1 | 0.71 | -11.27 |
| | Compare M3 and M2 | -0.04 | 1 | 0.27 | -41.72 |
| PSYTR | Compare M1 and M0 | 0.00 | 0.28 | -0.20 | 0.07 |
| | Compare M2 and M0 | 0.30 | 0.78 | 0.58 | 0.68 |
| | Compare M3 and M0 | 0.26 | 1 | 0.69 | -13.12 |
| | Compare M3 and M2 | -0.03 | 1 | 0.26 | -43.10 |
| NET | Compare M1 and M0 | 0.04 | 0.01 | -0.01 | 0.00 |
| | Compare M2 and M0 | 0.01 | 0.04 | 0.00 | 0.10 |
| | Compare M3 and M0 | 0.06 | 0.06 | -0.01 | 0.08 |
| | Compare M3 and M2 | 0.05 | 0.02 | -0.01 | -0.03 |

*Table 6 continues*

**TABLE 6 (continued)**

| | | Residual (Within Individual) | Intercept Variance | Time Variance | Intercept-Time Variance |
|---|---|---|---|---|---|
| DAMAG | Compare M1 and M0 | 0.07 | 0.27 | 0.21 | 0.04 |
| | Compare M2 and M0 | 0.09 | 0.20 | 0.08 | 0.15 |
| | Compare M3 and M0 | 0.14 | 0.42 | 0.22 | 0.19 |
| | Compare M3 and M2 | 0.06 | 0.27 | 0.15 | 0.04 |
| FOUL | Compare M1 and M0 | 0.03 | 0.01 | 0.01 | 0.01 |
| | Compare M2 and M0 | 0.03 | 0.05 | 0.03 | 0.67 |
| | Compare M3 and M0 | 0.06 | 0.07 | 0.04 | 0.46 |
| | Compare M3 and M2 | 0.03 | 0.02 | 0.01 | -0.66 |
| STOH | Compare M1 and M0 | 0.10 | 0.11 | 0.09 | 0.42 |
| | Compare M2 and M0 | 0.18 | 0.16 | 0.19 | 0.62 |
| | Compare M3 and M0 | 0.22 | 0.33 | 0.16 | 0.71 |
| | Compare M3 and M2 | 0.04 | 0.20 | -0.04 | 0.24 |
| TRESP | Compare M1 and M0 | 0.11 | 0.60 | -0.26 | 0.29 |
| | Compare M2 and M0 | 0.14 | 0.46 | -0.03 | 0.25 |
| | Compare M3 and M0 | 0.26 | 0.76 | -0.35 | 0.43 |
| | Compare M3 and M2 | 0.12 | 0.56 | -0.31 | 0.24 |

Note: M0 = Baseline model; M1 = Model 1, include predictor (group) with no covariates; M2 = Model 2, include two covariates (initial age and gender); M3 = Model 3, include predictor (group) and two covariates (initial age and gender). DELIN = scale score of the delinquency scale; DRUG = scale score of the substance abuse scale; SMOK = item score of smoking behavior; ALCO = item score of drinking alcohol; KETA = item score of using ketamine; CANNA = item score of using cannabis; PILL = item score of taking pills (e.g., ecstasy or methaqualone); HERN = item score of using heroin; PSYTR = composite score of illegal psychotropic drug use; NET = item score of ability to control Internet use; DAMAG = item score of destroying other's properties; FOUL = item score of speaking foul language; STOH = item score of staying outside the home overnight without parental approval; TRESP = item score of trespass.

predictor (group) added, M2 refers to the model with only two covariates (age and gender) added, and M3 refers to the full model with both predictor (group) and two covariates (age and gender) included. The column "Residual" reports the percent reduction in variance (within individual) between two models. The Pseudo-$R^2$ for "intercept" and "linear growth" is reported in the columns of "Intercept variance" and "Time variance", with the first model as the conditional model and the second model as the unconditional model. Pseudo-$R^2$ is a commonly used measure of effect size in linear multilevel modeling that considers the whole model effect. The value of Pseudo-$R^2$ represents the percentage of the variance in the variation of the intercept and the linear change in the dependent variable that can be explained by the added independent variable. For example, using M2 as the unconditional model and M3 as the conditional model, the results for ketamine use suggest that group explained 100% of the variance in the variation of intercept and 20.2% of the variance in variation of the linear change in ketamine use after controlling for the effects of initial age and gender. There are several observations based on the tables. First, for most indicators, group explained more than 13% of the variance in the variation of both intercept and linear growth, which according to Cohen's guideline (i.e., 0.02, 0.13, and 0.26 in $R^2$ change representing small, medium, and large effect, respectively) represent for medium to large effects of group on predicting the intercept and linear rate of change in the problem behaviors over time. Second, group explained about 3–27% of the within-individual variances. Third, some Pseudo-$R^2$ values were negative. This can be explained by the compensatory relation between the within-individual variance and between-individual variance[30]. More discussion about negative Pseudo-$R^2$ and alternative ways for calculating the $R^2$ for different level to prevent the negative explained variance can be seen in Snijders and Bosker's book[30]. In general, these findings are consistent with the tests of significance for individual parameter estimates as shown in Tables 3 and 4, and suggest that the effect sizes of group in predicting the linear growth in most problem behaviors are medium to large.

## DISCUSSION

The present study aimed to investigate the longitudinal effects of a positive youth development program, the Project P.A.T.H.S., on preventing youth problem behaviors with a randomized group trial. There are several unique strengths of this study. First, the present study had a very large sample size with more than 6,500 students participating in each wave of data collection, which ensured adequate power of the data analyses and permitted conclusions to be generalizable. Second, IGC modeling, as a superior data analytic method over traditional procedures, was employed to inspect the course of change of the outcome variables over time and the long-term effects of intervention. Third, multiple behavioral outcome indicators were examined. Fourth, as far as known, this study is the first attempt to evaluate the preventive effects of a positive youth development program on adolescent problem behaviors with a longitudinal randomized group trial in the context of Chinese culture. As suggested by Davis[31], the use of longitudinal and experimental design is the only way to establish causal relationship and delineate the process of how a program may impact the developmental trajectory of adolescent positive and negative outcomes. The present study illustrates the utilization of such a scientific approach in program evaluation.

Consistent with earlier findings, it was found that the occurrence of problem behaviors during adolescence increased with time. As expected, the rate of such a normative increase in delinquent behaviors (including the composite indicator of delinquency, and individual behavioral indicators such as speaking foul language, staying outside overnight without parental approval, and trespasses) and substance abuse (including ketamine, cannabis, psychotropic drugs, and heroin) was generally lower in the experimental group relative to the control group. Further comparison between the experimental participants who perceived the program as beneficial and the control group showed similar and stronger effects of the program. For example, apart from delinquent behaviors, students' self-control over Internet use also demonstrated significant program effect. While the control participants displayed a gradually deteriorating trend over time, the developmental trajectory of Internet use control for the experimental participants first showed a slower rate of decrease and then changed to a tendency of increase. These findings suggest that the Project P.A.T.H.S. may have effectively altered the risky developmental trajectories of adolescent problem behaviors for the participants and may have important implications for prevention practice.

Previous studies have reported that students who participated in the Project P.A.T.H.S. scored high on various positive youth development constructs, including positive identity, psychosocial competencies, resilience, bonding, recognition for positive behavior, and other high-order positive qualities than did students in the control group[19]. The promotion of positive youth development among the program participants is likely to contribute to the preventive effect of the Project P.A.T.H.S. on adolescent problem behaviors. Future research shall further establish the mediating or moderating effects of different positive youth development constructs in the prevention of problem behaviors with longitudinal studies. Some initial findings suggesting that life satisfaction is a mediating factor of the effect of positive youth development on adolescent problem behavior have been reported[32].

It is worth considering that program effects on problem behavior intentions were nonsignificant in the present study. In addition, the effect sizes of group on the growth parameters of different problem behaviors were relatively low. One possible reason might be that the present data on adolescent problem behavior had too many zero values and the zero value indicated not the lowest level on a continuum, but rather the absence of the behavior for which severity can be rated[33]. For example, for the composite score of substance abuse, the percentage of zero values at each wave were 73, 63, 63.1, 57.1, 57.9, and 54.5% (from Wave 1 to Wave 6), respectively, meaning that the problem of using drugs did not exist in more than 50% of the participants. For problem behavior intention, 61, 47.7, 48.5, 39.8, 41.1, and 36.6% of the participants at Wave 1 through Wave 6 reported absolutely no intention to engage in any delinquent or drug use behaviors in the coming 2 years. Under this circumstance, it has been suggested that using standard statistical methods that treat zeros as another point on the severity continuum may lead to under- or overestimation of effects and *p* values. For cross-sectional data, one solution is to analyze the zero values and nonzero values separately by using a two-part model[34,35]. However, for longitudinal data containing an abundance of zero values, there is currently no readily available method to address the time and program interaction issue. One way to further examine the effect of the Project P.A.T.H.S. on adolescent risk behavior and problem behavior intention is to compare the experimental group and control group at each separate time point in a cross-sectional fashion so that the two-part model can be used to provide more accurate estimation of the program effects. This could be another task in future studies. Another possible factor contributing to the nonsignificant effect is the use of a single item to assess behavioral intention to engage in a particular form of problem behavior. Such single items may not be able to capture the participants' real behavioral intentions fully. Measurement employing multiple items for the assessment of different types of problem behavior intentions should be developed and used in the future.

To sum up, findings of the present longitudinal study basically support the hypothesis that the Project P.A.T.H.S. is an effective approach to preventing youth problem behaviors. In conjunction with Shek and Ma's recent report[19] on the positive impact of the program on different positive youth development constructs over time and other evaluation studies[36,37,38,39,40,41], the current findings further evidenced the long-term effects of the Project P.A.T.H.S. Moreover, the present study demonstrated the use of IGC modeling in determining the longitudinal impact of a program, which may serve as a primer for future consideration of the IGC method in the field of program evaluation.
